# Tele-Education in South Africa

**DOI:** 10.3389/fpubh.2014.00173

**Published:** 2014-11-03

**Authors:** Maurice Mars

**Affiliations:** ^1^Department of TeleHealth, Nelson R Mandela School of Medicine, University of KwaZulu-Natal, Durban, South Africa

**Keywords:** tele-education, videoconference, South Africa, eHealth implementation, telemedicine, review

## Abstract

**Introduction:** Telemedicine includes the use of information and communication technology for education in the health sector, tele-education. Sub-Saharan Africa has an extreme shortage of health professionals and as a result, doctors to teach doctors and students. Tele-education has the potential to provide access to education both formal and continuing medical education. While the uptake of telemedicine in Africa is low, there are a number of successful and sustained tele-education programs. The aims of this study were (i) to review the literature on tele-education in South Africa, (ii) describe tele-education activities at the University of KwaZulu-Natal (UKZ-N) in South Africa, and (iii) review the development of these programs with respect to current thinking on eHealth project implementation.

**Method:** A literature review of tele-education in South Africa was undertaken. The development of the tele-education services at UKZ-N from 2001 to present is described. The approaches taken are compared with current teaching on eHealth implementation and a retrospective design-reality gap analysis is made.

**Results:** Tele-education has been in use in South Africa since the 1970s. Several forms of tele-education are in place at the medical schools and in some Provincial Departments of Health (DOH). Despite initial attempts by the National DOH, there are no national initiatives in tele-education. At UKZ-N, a tele-education service has been running since 2001 and appears to be sustainable and reaching maturity, with over 1,400 h of videoconferenced education offered per year. The service has expanded to offer videoconferenced education into Africa using different ways of delivering tele-education.

**Conclusion:** Tele-education has been used in different forms for many years in the health sector in South Africa. There is little hard evidence of its educational merit or economic worth. What it apparent is that it improves access to education and training in resource constrained settings. The development of local and international tele-education at the UKZ-N has not followed what is currently considered to be best practice but shows how programs can develop if there is a real need and the solution assists in meeting the need. Further work is required to analyze the economics of these tele-education endeavors.

## Introduction

The uptake of telemedicine in Africa has been slow. There are many reasons for this – the burden of disease, shortage of doctors, poverty, limited funding of health services, lack of infrastructure, poor but costly connectivity, irregular power supply, lack of political will, and limited computer literacy (1). Telemedicine also adds extra steps to the routine workflow, adding burden to already overworked health professionals. But telemedicine is not confined only to clinical services. The World Health Organization’s definition of telemedicine includes the use of information and communication technologies (ICTs) “for the continuing education of health-care providers.” (2). This meets the definition of tele-education, which Curran defined “as the application of ICTs in the delivery of distance learning” and noted that the way in which it is delivered is dependent on the technologies and media used (3).

Education is often used as a catch all word to encompass three distinct activities; formal education leading to an academic qualification, training, and acquisition of skills, and raising awareness. In this paper, tele-education will include education in its broadest sense.

One of the perceived benefits of telemedicine is overcoming the sense of isolation experienced by doctors in rural communities. This can be achieved by providing professional support through second opinion consultation, referral, access to specialists and specialist services, and continuing medical education. The benefit of tele-education to rural doctors has been described in Australia and North America (4, 5). The experience in Africa is similar, not only for doctors in rural areas but also doctors in urban areas where specialists may be in short supply. There are several examples of successful and sustained educational activities.

The Reseau en Afrique Francophone pour la Telemedicine (RAFT) program initiated by the University of Geneva in 2001 currently links up to 18 countries in Francophone Africa using low bandwidth videoconferencing (VC). It provides 16 h of education a month with most of the teaching emanating from African countries (6). The Institute of Tropical Medicine in Antwerp uses email, the Web, and mobile phones to provide HIV education (7), and Medical Missions to Children provides daily seminars broadcast three times a day via satellite and the Internet (8). The Pan-African eNetwork between India and many African countries (9), African Medical Research Foundation (AMREF), which is reskilling nurses in central and East Africa (10), and Project Hope providing HIV training between the US and five African countries are other examples of sustained educational activities in Africa (11). Unlike the developed world, much of this education takes place in urban rather than rural areas because of the limited telecommunication infrastructure in rural Africa.

The WHO definition of telemedicine refers to continuing medical education, but what of its role in graduate and undergraduate education?

The extreme shortage of doctors in sub-Saharan Africa is well documented with 26 doctors per 100,000 people against a global average of 141 per 100,000 people and 331 per 100,000 people in the European Region of the WHO (12). Three approaches have been proposed to address the shortage; increase the number of medical graduates, make migration of doctors from the developing world more difficult, and train medical assistants to perform certain tasks.

The number of medical school in sub-Saharan Africa has increased rapidly over the past 15 years. In 2000, the World Directory of Medical Schools recorded 77 medical schools in sub-Saharan Africa (13). By 2006, there were 86 medical schools (14) and the sub-Saharan Africa Medical School Study of 2011 found 169 medical schools, several of which were not yet graduating students (15). The Foundation for Advancement of International Medical Education and Research, which maintains the International Medical Education Directory lists 122 medical schools that are active and recognized by the appropriate Ministry of the country (16). With the extreme shortage of doctors, who is educating and training students in these new and developing medical schools, especially in the specialties?

The situation is no different in South Africa. While South Africa has 77 doctors per 100,000 people this includes, retired and non-practicing doctors and those who have remained on the register but practice in other countries (12) and it is estimated that the actual figure is nearer 55 doctors per 100,000 people (17). There are 39,800 medical practitioners registered with the Health Professions Council of South Africa (HPCSA) and an additional 14,000 doctors are required to merely fill the vacant posts in the Government sector (18). The eight South African medical schools produce around 1,300 doctors per annum. A new medical school is planned but none have been built since the 1970s when the population of the country was less than half its present number. Doubling the output of the eight medical schools in South Africa and opening a new medical school will merely maintain the current doctor to patient ratio (19). To overcome this, medical schools are increasing their intake but are constrained by physical, human, and financial resources and the number of “teaching beds” in academic hospitals. The government has also been sending students from rural areas to Cuba for medical training and plans to increase the number sent and graduating to 1,000 over the next 5 years (20). A new category of practitioner, clinical associate, is being trained at four of the medical schools to work in district hospitals under supervision of a physician. This is a 3-year bachelor degree program.

In the African and South African context, the WHO’s definition of telemedicine needs to be expanded to include undergraduate professional development as it improves access to scarce academic resources.

The aims of this study were to (i) review the literature on tele-education in South Africa, (ii) describe tele-education activities at the University of KwaZulu-Natal (UKZ-N) in South Africa, and (iii) review the development of these programs with respect to current thinking on eHealth project implementation.

## Materials and Methods

### Literature review

A literature review was undertaken of telemedicine in South Africa. Searches were conducted of Pubmed, Scopus, Cinahl, African Journals online, and African Index Medicus using search terms such as ((“South Africa”) AND (“tele-education” OR “distance education” OR “videoconference” OR “remote education”)). As tele-education falls under the definition of telemedicine the databases were also searched using common keywords relating to telemedicine. For Pubmed, the terms ((“telemedicine” OR “ehealth” OR “mhealth” OR “email” OR “electronic mail” OR “telephone” OR “mobile phone” OR “cell phone” OR “videoconference”) AND (“South Africa”) AND (“tele-education” OR “distance education” OR “remote education”)) were used to search the title, abstract, and keywords of articles indexed. Google Scholar and Google were searched for each of the eight medical schools and nine Provincial Department of Health (DOH) for VC or tele-education.

The abstracts of all papers returned by the searches were read. The full papers of those that appeared relevant were obtained and read. Those reporting the use of ICTs for education in the health sector in South Africa were included in the review. The aim of the review was to document evidence of tele-education in South Africa and no attempt was made to assess the quality of the papers.

### Case study

A single case study was undertaken of tele-education at the UKZ-N. The objective of the study was to describe the evolution of the service and compare its development with current thinking about eHealth implementation. The records, reports, and minutes of meetings related of the service were reviewed.

### Design-reality gap assessment

A retrospective assessment of the design-reality gap at the time of implementation of videoconferenced tele-education at the UKZ-N was undertaken using Heeks’ Design-Reality Gap Assessment model (21).

## Results and Discussion

### Literature review

Eighty-three papers and Web pages relating to tele-education in South Africa were found, of which 23 were relevant and summarized. Of these, 16 reported tele-education activities, 5 provided data on tele-education, and 2 referred to the educational benefits of participation in a clinical telemedicine service.

Tele-education in South Africa has followed the evolution of technology and has taken many forms. It can be broadly categorized in terms of continuing medical education, postgraduate education, undergraduate education, and education through participation in telemedicine services.

In the 1970s the South African College of Medicine provided continuing medical education using tape recordings of lectures (22). As South Africa was a late adopter of television in 1975, there was little use of closed circuit television for education, as pioneered in Nebraska for mental health in the 1950s (23). Wessels noted the educational value to the general populations of a television series that focused mental health in 1999 (24). The use of tele-education in nursing and medical courses is not new in South Africa with nursing courses being offered by satellite as early as 1990.

The South African government saw the potential of telemedicine and implemented a National Telemedicine System in 1999. It was to have consisted of three phases. The first, pilot phase, involved establishing telemedicine services at 29 sites in six of the nine provinces. The second phase envisaged connecting the eight medical schools by VC for the provision of both postgraduate and continuing medical education and extending the original project to 76 VC sites. Although there were promising early reports of the system, only a teleradiology store and forward service in neurosurgery was sustained, with most services failing within 2 years (25, 26). As a result, phases two and three were never implemented. In the absence of a Government backed telemedicine system, various Provincial DOHs, Universities, and the Medical Research Council of South Africa continued to support and develop telemedicine and tele-education.

Part of the problem appears to have been that the National Telemedicine System was conceived, planned, and budgeted centrally in the National DOH, with the National Health Information System of South Africa (NHISSA) Committee having oversight. After the installation of equipment in phase one, the Provincial DOHs were expected to take ownership of “their” programs and submit monthly reports to NHISSA. Telemedicine was an additional function within the Health Information units in the Provinces with no budget or posts for telemedicine in place (27).

In Free State Province, the Provincial DOH has used an interactive-learning and communication and management network (ICAM) since 2002 with initial setup costs of ZAR11 million (US$ 2 million at that time) and a further ZAR12 million spent to upgrade facilities in 2011. The Department currently runs a broadcasting facility including three control rooms, three broadcasting studios, two editing rooms with voiceover booths, and editing equipment. Educational content, lectures, seminars, and practical training sessions presented by DOH staff and academics from the University of the Free State Medical School are filmed, recorded, edited, and broadcast by satellite link to 40 sites in the Province. Two way audio communication is provided through a response keypad. There are approximately 16,000 participants in these sessions annually (28).

At the University of Stellenbosch interactive television was used in the early 2000s to see and hear lecturers, with students interacting by telephone from the receiving studio (29, 30). The Ukwanda Rural Clinical School has a telemedicine and eHealth component with a VC link between the medical school and two rural health-care hostels allowing undergraduate students to receive education while on rotation in rural areas (31). Several postgraduate programs in nutrition and reproductive biology and 11 in nursing are offered at a distance by VC with the University having access to 19 VC venues across the country (32).

In 2006, a nationwide radiology education program for trainees was trialed, initially between three medical schools and later with seven participating via a commercial VC bridge service. Problems were noted with the quality of the radiographic images shared over the network as connectivity defaulted to that of the site with the lowest bandwidth, 128 kbps. To overcome this, images of radiographs were saved to PowerPoint and distributed by email before the scheduled sessions and projected locally with VC used for discussion between the sites (33). A plan to establish shared postgraduate radiology teaching nationally never materialized.

In the Eastern Cape, telemedicine services were initiated in the late 1990s and tele-education by VC was reported in 2001 (34). Banach reported education as part of the Eastern Cape telemedicine activity in 2008 with four health resource centers linked for teaching and case presentation (35).

At the University of Cape Town, the Department of Pediatric Surgery has installed video cameras in their operating theaters so that surgical procedures can be broadcast to lecture theaters (36). They have recently begun sharing weekly seminars with some medical schools in Africa using Internet protocol (IP) based desktop VC. The Department of Gastro-enterology has participated with the Universities of the Witwaterstrand and KwaZulu-Natal in receiving high definition video of endoscopic surgery undertaken and streamed from Japan (37). In 2011, a working group was formed to investigate the use of VC in education at the University and a VC link has been established with a rural hospital.

The University of South Africa is a distance learning university with 19 VC venues in South Africa. No information is available on tele-education in the health sector which includes nursing and public health.

The Mindset Network is a non-profit organization in partnership with the National Departments of Education, Health, Communication, Science and Technology, and communication companies. Started in 2002, it provides educational material to school children – Mindset Learn, teachers – Mindset Teach and the health sector – Mindset Health through digital satellite television channels. Mindset Health provides recorded programing of health related issues for patients and the public which is played in waiting areas of clinics and public hospitals. These are provided via subscription free digital satellite television through Sentech and Intelsat, with equipment installations funded by donors.

The health content covers topics such as prevention of mother to child transmission of HIV and infection prevention and control delivered in the form of dramas, discussions, interviews, documentaries, and magazine shows with content aligned to the National DOH policies and guidelines. The Mindset outreach program provides training to health workers on facilitating use of the Mindset Health video content for formal and informal training in clinics and other community settings. Currently, the Mindset Health Channel is available in 625 public health facilities in the country with a further 788 sites planned as part of the implementation of the National Health Insurance in South Africa. Material is also available on YouTube but use of this facility has been very limited.

Mindset provides an online certificate course, on the Fundamental Management of HIV and Aids for health-care providers, using a learning management system. The course is integrated into the curriculum of nursing schools and is used for continuing professional development. No data are available evaluating use of Mindset Health (38).

After the failure of phase one of the National Telemedicine System in KwaZulu-Natal, the University of Natal used the VC equipment for tele-education and videoconferenced education in radiology began in 2001 with point to point VC at 128 kbps (27). By 2010, this had grown to 35 academic programs broadcasting a total of 123 h of interactive teaching per month with 37 different sites participating in the various programs (39).

In pediatric surgery, weekly seminars have been shared at various times with an academic hospital in the Eastern Cape, a regional hospital in Limpopo Province, neither of which had a pediatric surgeon and recently an academic hospital in Zimbabwe. In the first 4 years of the service the seminar, attended in Durban by an average 13 people, was shared with 63 others at the various sites (40). Surgeons from several other African countries have requested to be included in the videoconference program. In the absence of VC infrastructure at other medical schools in Africa, the VC seminars in Durban have been recorded to digital video disk (DVD) and mailed to four medical schools in Central and East Africa. They have been incorporated into the postgraduate training programs of surgeons and pediatric surgeons and also used in undergraduate medical training. An additional 140 people have access to the seminars through this program (41).

Chipps et al. conducted a systematic review of the effectiveness VC-based tele-education for doctors and nurses and reported equivalence with face to face teaching and an increase in knowledge and knowledge retention reported in one study (42). Videoconferenced teaching for psychiatry registrars was then implemented with 6.5 h of VC education conducted weekly using ISDN connections at 128 kpbs. Over the period of review, there was a significant increase in the number of trainees who elected to join by VC and not travel to the Medical School with concomitant savings in time and transport costs. Audio quality, while satisfactory during presentations was a problem for some during discussions. Participants were satisfied with the quality of PowerPoint presentations but concerns were raised about some teaching aids, notably problems with the colors in drawings. Both local and distant attendees were less satisfied with the use and visualization of specimens in anatomy sessions (43).

To extend mental health education outreach, a set of lectures was videoconferenced to doctors at designated Mental Health Hospitals in KZ-N at 128 kbps. As it was difficult to co-ordinate activities at the different hospitals, the lectures were subsequently recorded to DVD and sent to the hospitals for local use. Pre and post testing of participants’ knowledge showed improvements in post test scores (44). A pilot project assessed the feasibility of teaching nurses taking a decentralized educational program by VC and this was later adopted (45).

The American Medical Informatics Association developed the concept of HIBBS, health informatics building blocks, in their approach to increasing the number of informaticians. The HIBBs program aims to create a variety of training modules on health informatics that may be used as open educational resources. Under the direction of the Global Health Informatics Partnership, four HIBBS were developed at UKZ-N, assessed by senior academic informaticians in the US, and made available on the Internet through Open Educational Resources Africa (OER Africa). Each module consists of a PowerPoint presentation, an audio file, a video of the presentation with audio, and a Word document with the full text of the presentation. Pre and post testing with repeat post testing after 6 weeks showed significant improvement in both post test scores *p* < 0.001. The University of Cape Town, a founding partner of OER Africa has made a large number of educational videos and presentations available on the site.

The educational benefits to doctors and nurses participating in clinical telemedicine service have been documented. An early audit of a synchronous teledermatology service in KZ-N evaluated the educational benefit of being able to discuss cases with a dermatologist with the educational value of 86% of teleconsultations rated as good or very good (46). In a store and forward tele-ophthalmology service, it was noted over time that only more complex cases were referred with the referring doctor having learned from the responses obtained from the ophthalmologists for previous cases (27).

Colven quantified this in a study of a store and forward teledermatology service between five primary care physicians and one dermatology trained nurse at five rural sites and the Department of Dermatology at the University of Cape Town. In addition to the diagnosis and management plan, referrers were provided with relevant references. Correlation of diagnostic concordance between the referrer and the dermatologist and the number of referrals over time was significant and increased from 13% for the first four referrals to 50% for the 9th to 12th referrals. Partial concordance, the dermatologists diagnosis included in the referrers’ differential diagnosis, also increased significantly over time from 33 to 60% (47). The number of referrals per month decreased significantly over time, and it is not clear whether this was due to improved diagnostic acumen resulting from the service and references provided (48). It was noted that although a clinical referral template was available this was used only 27% of the time, and mostly by the only nurse referrer with doctors including the history in the body of the email sent. Over the 29 months of the service, 4.1 cases were sent per month, which equates to less than one case per site month. This is in keeping with the finding that 61% of telemedicine services world-wide refer fewer than one case per site per week (49).

#### mHealth

The GSMA website lists 96 mHealth projects in South Africa but none appear to be offering clinical services other than call center advice. There are many examples of SMS services for patient education, appointment reminders, treatment adherence, and patient support.

Woods et al. (50) describe the use of short message service (SMS) text messages for providing ongoing education to midwives. The service was linked to a Website from which additional reading material could be downloaded. The service was well received but only 17 (34%) of 50 interviewees accessed the Website, while 16 (32%) indicated that they wished to access the Website but did not have Internet access (50). This highlights the problems faced in delivering IP based educational material to rural areas. While an assumption is made that smart phone technology will address the problem, the reality is that cellular phone service providers are reluctant to deploy expensive infrastructure for poor people in rural areas who are not able to afford the service.

#### Regulatory issues

The HPCSA is a statutory body tasked with licensing health professionals, providing guidelines for ethical practice and “to serve and protect the public in matters involving the rendering of health services by persons practicing a health profession.” The HPCSA has been working on General Ethical Guidelines for Good Practice in Telemedicine for over 7 years. The most recently available draft version defines telemedicine incorrectly as, “*the exchange of information on health care at a distance for the purpose of facilitating, improving and enhancing, clinical, educational and scientific health care and research, particularly to the under-serviced areas in the Republic of South Africa*” (51). This definition fails to include the use of information and communication technology. As the guidelines propose that written informed consent be required for all aspects of a telemedicine encounter with copies kept by both the provider and recipient, this would include tele-education and will add unnecessary administrative load to those providing tele-education (52).

### Tele-education in KwaZulu-natal – a case study

Videoconferenced tele-education began in KwaZulu-Natal in 2001. The evolution of this service will be described and then compared with current thinking about eHealth implementation and project risk.

The proposed National Telemedicine System planned to include tele-education in its second phase with the expectation that the eight medical schools would offer shared postgraduate teaching and continuing medical education. An education sub-committee of the National Telemedicine System Committee was formed and tasked with facilitating tele-education. As the second phase was never funded, the medical schools did not receive VC units, nothing came of the plan and the committee was disbanded.

As a member of the sub-committee, the author saw the potential of tele-education to address, in part, some issues facing postgraduate education at the medical school in the University of Natal. The medical school is situated in Durban and has satellite teaching hospitals in the Durban region and several towns up to 300 km away. These hospitals are accredited to provide trainee specialists, registrars/residents, with a portion of their supervised clinical training time. The postgraduate academic programs of all specialties at the medical school include weekly seminars, in many instances weekly journal clubs and research meetings and in radiology, daily teaching sessions, all of which are conducted at the medical school. Staff at distant hospitals find it difficult to attend because of the effect of traveling and participation time on clinical services. Videoconferenced tele-education was seen as a potential solution. In effect, the solution to the problem was proposed without a formal needs assessment.

In KwaZulu-Natal, nine VC sites were established for tele-ultrasonography and tele-ophthalmology in Phase One of the National Telemedicine System. Site visits were undertaken to determine whether staff at these sites envisaged developing telemedicine services using VC. No services were planned and at several sites, the units had been disconnected and stored. Permission was sought from the Provincial Member of the Executive Committee for Health for the medical school to relocate some of the VC units and use them for post graduate tele-education.

A decision was made to begin on a small scale, learn from the experience, refine the approach, and then scale up services. The heads of two clinical departments, surgery, and radiology were approached, asked to relocate their teaching venues to VC venues and broadcast their teaching sessions to another hospital.

At the outset, a simple principle for the provision of educational material for tele-education was adopted: no-one must do extra work. In the absence of software to transmit a PowerPoint presentation from a computer to the VC unit, the camera in the seminar venue was aimed at the screen on which the presentation was being projected. As technology improved, it was possible to send the presentations directly through the VC unit.

All training support was provided by local technicians. For the first 4–6 weeks of linking to a new site, a technician traveled to the site and ensured that the connections were made and trained local doctors to make connections and perform basic troubleshooting. Support from Durban is also provided by telephone during every VC session. Presenters were encouraged to ensure that participants at distant sites were drawn into discussions and asked questions. PowerPoint presentations posed an unforeseen problem when there were too many lines of text on a slide, fonts were too small and the contrast between text and background was poor. Although guidelines were developed and given to presenters, few complied. The technician attending the session would request the presentation a day in advance and make changes as needed. This was not without its problems.

The number of sites able to participate in a session was limited by the available software and technology, which initially limited it to the send site and one other site. As VC equipment and software improved, it became possible to link to three additional sites and then to five sites. In the absence of a VC bridge, units with multisite capacity were “daisy chained” so that a five port site could use one or more of its ports to link to other three or five port sites, which in turn linked to other sites.

**Figure 1:**
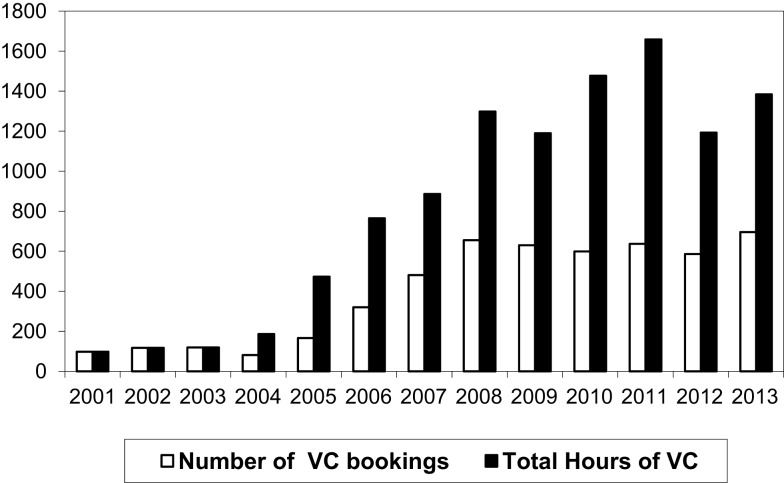
**The number of hours of videoconferenced teaching and number of videoconferencing bookings per year from 2001 to 2013**.

Videoconferenced tele-education grew as per Rogers’ diffusion of innovation theory (Figure 1).

**Figure 2:**
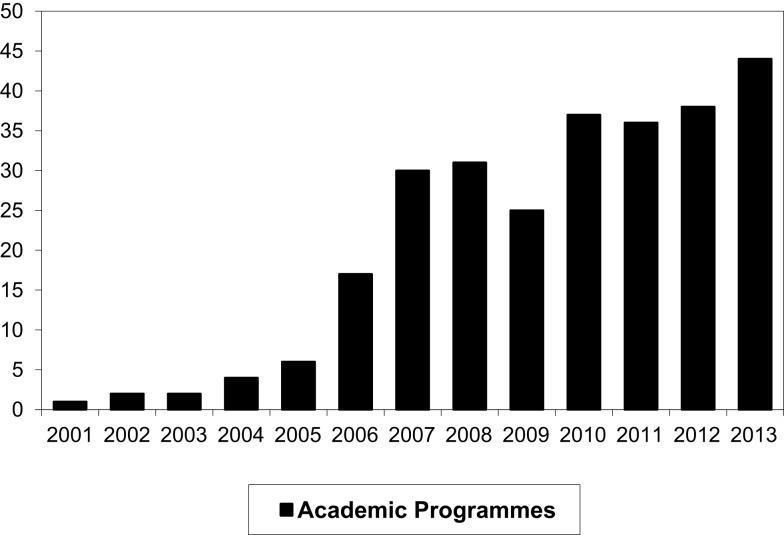
**The number of academic programs and activities using videoconferencing for teaching**.

The number of programs and activities participating in VC teaching has increased and is nearing saturation (Figure 2).

In 2005, a postgraduate program in HIV Management was the first academic program designed to be delivered by VC. Lectures were given at the Medical school linked to four regional hospitals in the province. Students went to the nearest site and participated in classes that ran over weekends. Administrative support was provided at each site.

The original VC units installed during the National Telemedicine System were Polycom units with connectivity provided by ISDN lines at 256 kbps. Subsequent installations were at 128 kbps for distant sites and 384 or 512 kbps for multisite units. Use of ISDN was dictated initially by VC equipment and access to bandwidth. As IP based VC units and software became available, ISDN continued to be used because lack of IP bandwidth at hospitals. Until recently, most hospitals in the Province were provided with 128 kbps bandwidth shared between administrative and clinical services.

#### Administration

Preceding the National Telemedicine System, the then university of Natal and KZ-N DOH formed a joint committee to work on the ICT needs of both sectors at the planned Inkosi Albert Luthuli Central Hospital, a paperless hospital developed under a public private partnership. This served as the precursor to the KZ-N Telemedicine Steering Committee. When the KZ-N component of the National Telemedicine System was devolved to KZ-N DOH, they were expected to administer and submit monthly reports on their telemedicine activity. As the KZ-N DOH did not have a budget for telemedicine and were not organizing telemedicine services the University assumed these functions.

This led to the Joint KZ-N DOH and UKZ-N Telemedicine Steering Committee made up of the IT Manager of the DOH, the three area managers of the Province and three representatives from the Medical School, staff in the Department of TeleHealth. The administrative model was fraught with problems. The KZ-N DOH had not taken ownership of telemedicine or tele-education. Telemedicine was an additional responsibility for the managers concerned and without a budget they had little that they could add of value. While the three area managers understood that the DOH was supportive of telemedicine they had little understanding of what telemedicine entailed in terms of infrastructure, staff and administration. Between 2002 and 2010, awareness sessions were arranged within each of the three areas so that the 11 district managers and the hospital managers would understand why ICT infrastructure had been installed and what telemedicine was planned for their hospitals.

Subsequent studies by Chipps et al. showed limited understanding of both area managers and district managers of what was being planned for telemedicine even though the infrastructure was already in place (53). Few were aware that tele-education was taking place within their jurisdictions.

The Telemedicine Steering Committee met on a quarterly basis for several years and endorsed activities planned and run by the University. Managers, however, lost interest in the committee as it had no budget and there were no posts and people to do the work within the DOH. They also did not see tele-education as part of telemedicine. The situation has recently improved with the appointment of a Telemedicine Manager and four support staff. Their focus remains clinical telemedicine and they do not have a dedicated budget.

#### Provision of infrastructure

Additional VC units have been installed over the past 8 years through funding from the Global Fund and a co-operative agreement between the Italian Government and the KZ-N DOH. Each of the 11 districts has at least three units and there are currently over 45 VC units in the province with several at the main teaching hospitals. Each VC unit has a data projector, computer, sound system, and security. Despite security cages, locks and burglar bars, several units, and sound equipment have been stolen. It was agreed that the University would maintain equipment at teaching hospitals and the KZ-N DOH the remainder. No procurement, maintenance or replacement budgets exist, and replacement is dealt with on an *ad hoc* basis.

The need to continue using ISDN for connectivity remains a problem especially in rural areas. The national telecommunications company, Telkom, is the sole provider of ISDN and although the Provincial DOH receives priority maintenance this has not always been forthcoming. On a number of occasions, one line at a site has become faulty and can take several months to be fixed. Services have also been disrupted when hospital administrators do not realize that VC is by phone line and fail to pay the phone line rental bills.

#### Support

Onsite technical support for VC is another unresolved problem at the hospitals. Following current best practice of the day in 2000, super users were appointed at each hospital. They ranged from secretarial staff to a store foreman. IT support and VC support was not within their job descriptions and their first priority was to their line manager and allocated work. The Department of Health was not able to provide training on the VC units for the super users. This was done by technicians from the Department of TeleHealth.

The need for dedicated site co-ordinators based on the Canadian model was soon identified. It was suggested that they be nursing staff and that a percentage of their time be allocated to videoconference support for both educational and clinical activities. As nurses work different shift hours and because some tele-education sessions occur before and after normal work hours and over weekends, it was proposed that three nurses be appointed at each site with up to 25% of their time allocated to telemedicine support. This was agreed to by the IT Steering Committee in 2008 and job descriptions were drawn up. The Provincial DOH’s human resources division has yet to implement this.

#### Local administration

The administration of tele-education within the University has changed over the years. Initially, the medical school had a computer committee that reported to the Faculty Administrative Committee. This committee met regularly with the University IT Division and co-ordinated infrastructure installation and the development of wireless links between teaching hospitals. The Department of TeleHealth was formed in 2002 as an academic department, which also provides support for tele-education and telemedicine. The head of department and a newly appointed Faculty IT Multimedia Manager reported directly to the Dean of the faculty. Two technicians support the IT Multimedia Manager and all work out of the Department of TeleHealth. After restructuring within the university, the administration of tele-education now rests with the Faculty IT Multimedia Manager.

#### Fogarty International Training Grant Model and International Tele-education

The University of Natal was the local partner with Tufts University in Boston in an international training grant to educate people in sub-Saharan Africa in medical informatics and research in this field, which started in 1999. The program followed the then standard model of sending students and staff to the US for specific courses or to complete masters degrees and bringing faculty from the US to provide short courses. While meeting the expectations of the funders, those who were successful returned to unsupportive work environments and found themselves either working in isolation or unable to use their new knowledge. After the first cycle of funding the model was refined.

A postgraduate Medical Informatics program was established at the newly merged UKZ-N. Instead of sending students to the United States two or three students from a country were brought to South Africa for their postgraduate education. This enabled more students to benefit from the program but still took productive workers out of the workforce in their country. In the third cycle of funding, the model was further refined to take advantage of advances in ICT and improving bandwidth in African countries. Students were enrolled in several African countries and undertake their studies part-time while staying and working at home. A further aspect of the program is to develop capacity among staff within local universities in the field of medical informatics within local Universities so that in time they will be able to participate in teaching courses on the program with the end goal being to ultimately establish Medical Informatics Departments in their home institutions – “building the capacity to build capacity” (39). The program looks to educate 100 people across Africa.

This changed the approach to tele-education. No longer was teaching taking place by VC within KZ-N or to sites outside the Province via ISDN links. Attempts were made to convert ISDN input and output to IP using commercially available converters. This was not successful. A mixed mode approach to connectivity was needed. The first step was to adopt a learning management system to facilitate among other things, distribution of teaching materials, return and marking of assignments, interactive discussions and delivery, and return of examination papers and scripts. As a developing world country, it was decided to follow the Government’s policy of using an open source software. After reviewing the literature, Moodle^®^ was chosen and later adopted for use throughout the University.

An open source, IP, web-based VC was required to enable participation by students at sites that did not have ISDN connectivity. While Skype^®^ had evolved to a stable one to one link, options like NetMeeting were not yet available. DimDim^®^, a product developed in India offered multisite IP based VC in a Skype^®^ like environment. This free, open source solution integrated with Moodle^®^ allowing scheduling within Moodle^®^. However, it required all connections to go through its server in India. For multisite connections from South Africa, a latency of up to 7 s was noted. There was also a differential latency between audio and video such that audio transmission was faster than video and when lecturing and changing a PowerPoint slide the audio preceded the video to the point that that the sound could be several slides ahead of what was seen. A commercial license was obtained to mount the DimDim^®^ software on a local server, but this proved to be unstable and was abandoned.

The approach taken within the international training grant was to build capacity with students situated locally and outside of South Africa. This meant that there were students who were physically present at lectures and seminars in Durban, students at other sites in South Africa, and students at several sites in Africa. The problem was how to enable screen sharing and interactive audio communication between sites connecting using ISDN based and IP communication. The solution was simply to run the two systems in parallel. One computer was linked to the ISDN based videoconference unit. A second computer ran Skype^®^ with a multisite license over the Internet with students at distant sites initially connecting through their local service provider who connected via satellite.

The initial experience was with the Kigali Institute for Health in Rwanda. At the far site students gathered in a venue with an Internet connection and linked their computer to a data projector so that they could see the presenter on a shared screen. Audio was amplified through desktop speakers, one of the students controlled a Webcam, aiming it at whoever was speaking and a cheap off the shelf and an omni-directional microphone on a long lead was passed around between participants as and when required. This approach required the presenter to remember that a dual system was in use.

For the people at the different sites to hear and communicate with each other, judicious placement of speakers, and microphones on a table in front of the presenter was required, so that audio from the Skype^®^ participants was directed to the microphone of the ISDN participants and vice versa. Surprisingly, audio feedback was rarely encountered. Skype^®^ proved to be a simple solution and as it developed into multi-conferencing, it was used to connect several sites. Skype^®^ chat was also used to allow people to pose questions during presentations. Another solution available at the time but not used was Elluminate^®^ as this requires a commercial license and our approach was to use open source or shareware solutions when and where-ever possible.

Screen sharing of presentations, demonstrations of software, and coding exercises presented another problem because of latency when using Skype. Different approaches were taken. One solution was to save PowerPoint presentations to Moodle^®^ and have the presentation shown locally via a data projector to the assembled students. With limited bandwidth and high costs of data download, files of over five Meg proved difficult and costly to download. An alternative was to save the PowerPoint presentation as a handout with six slides to a page and save this in Adobe pdf format, which substantially reduces the file size. One of the students at a far site would show the appropriate slide locally using Adobe Reader.

Another use of VC that was not envisaged is its use for invigilating examinations.

A VC bridge was acquired by the Medical School through another Fogarty International Training Grant (MEPI). This enables up to 48 sites to be connected simultaneously but only through the Internet. To include ISDN sites, one of the multisite VC units has to connect to the bridge via IP and then connect to the ISDN sites. The KZ-N DOH has also acquired a VC bridge, which has not been used for tele-education.

#### Reflection

The local tele-education service and international program has evolved over time and adapted to changing needs, circumstances, and technology. It has grown and appears to be sustainable. When viewed in terms of current thinking about planning of eHealth projects few of the processes that are currently felt to be correct were undertaken.

It is said that there should be an eHealth strategy in place for successful introduction of eHealth programs (54). While the country had a telemedicine strategy, the telemedicine program had failed. Neither the University nor the KZ-N DOH had a tele-education strategy. The University did have a strategy for the use of VC for administrative functions and meetings between its various campuses.

The problem of providing teaching to off-site trainees was well known and no formal needs assessment was made. A comparison of possible solutions was not made and the decision to use VC was made unilaterally based on circumstantial availability of VC equipment and connectivity. No assessment of human and financial resources was made. The decision to adopt the principles of no-one doing any extra work and starting small and learning form experience reduced human resource issues to technical and administrative support with the University providing the support. No formal business plan was developed. At the outset the KZ-N DOH supplied equipment that was not being used so no direct cost was incurred and agreed to pay for the ISDN line rentals and the call costs for the educational sessions. The University provided seed funding to set up a VC site at the medical school and provided the time of the technical staff. The regulatory environment was not considered to be an issue as there were no regulations relating to telemedicine or tele-education.

An eReadiness assessment was not undertaken as the infrastructure was in place and it was acknowledged that training on the use of the equipment would be required. A shortcoming at this stage was that while trainees were made aware of the services, hospital managers did not necessarily buy-in to the project. A fundamental implementation plan that included a change management plan was developed for each new site that was used. Growth of the number of programs participating was due largely to observation of other departments doing it. Although not formalized, each of the programmes participating in the first five years was evaluated at various times for user satisfaction and technical issues. No formal economic assessment has been made although rudimentary assessment was made of psychiatry teaching (43).

### Design-reality gap assessment

No risk assessment was made during the planning phase. This has been undertaken now, after the fact, in an attempt to assess the risk at the time. Design-reality gap assessment that evaluates seven dimensions, information, technology, processes, objectives and values, staffing and skills, management systems and structures, and other resources has been used as it is a relatively simple method (21). The seven dimensions are scored from 0 to 10 with zero reflecting no change between the design proposal and current reality (Table 1).

**Table 1:** **Design-reality gap assessment showing the question asked, score, and answer**.

Question	Score	Answer
**Information:** What is the gap between the information assumptions/requirements of the new e-learning system design, and the information currently in use in reality in the Hospital?	1	The information provided by e-learning is the same as currently used. Some slide presentations may need to be edited.
**Technology:** What is the gap between the technology assumptions/requirements of the new e-learning system design, and the technology currently in use in reality in the Hospital?	7	The technology is in place, but staff are not trained or used to it.
**Processes:** What is the gap between the work processes required for successful implementation of the new e-learning system, and the work processes currently in use in reality in the Hospital?	2	There does not appear to be a gap between the current processes; however, participants at distant sites may feel excluded by not being face to face and may be excluded from discussion.
**Objectives and values:** What is the gap between the objectives and values that key stakeholders require for successful implementation of the new e-learning system design, and their current, real objectives, and values?	1	There needs to be acceptance of VC. Some people may be technophobic. The objectives of key stakeholders are the same.
**Staffing and skills:** What is the gap between the staffing numbers and skills levels/types required for successful implementation of the new e-learning system design, and current, real staffing, and skills?	7	There are no local and support staff at the distant sites and doctors and nurses have no technical training. It is assumed that after training staff will be able to make connections and undertake basic trouble shooting with support from Durban.
**Management systems and structures:** What is the gap between the management systems and structures required for successful implementation of the new e-learning system design, and current, real management systems, and structures?	5	Management systems are in place for delivery of educational sessions but participation at the far sites is dependent on local managers employed by the DOH and on the Provincial DOH continuing to sanction VC e-Learning.
**Other resources:** What is the gap between the other resources (money, time, other) required for successful implementation of the new e-procurement system design, and current, real availability of those resources?	5	The University has the resources to maintain and support the system at its venues but is dependent on the DOH for continued support at many of the distant sites.
**Total**	28	

The score of 28 places it on the border line between a project that might be a partial failure and an e-health project that might fail totally, or might well be a partial failure unless action is taken to close design-reality gaps. A score of 14 or less suggests success. Despite the seeming lack of planning for the tele-education projects, the risks were not perhaps as great as might be expected.

## Conclusion

Tele-education has been used in different forms in the health sector in South Africa for a number of years. There is little hard evidence of its educational merit or economic worth. What is apparent is that it improves access to education and training in resource constrained settings. The development of local and international tele-education at the UKZ-N has not followed what is currently considered to be best practice but shows how programs can develop if there is a real need and the solution assists in meeting the need. Further work is required to analyze the economics of these tele-education endeavors.

## Conflict of Interest Statement

The institution received payment from the Global Partnership Program for the development of four Health Informatics Building Blocks.
